# Modulating cholesterol-rich lipid rafts to disrupt influenza A virus infection

**DOI:** 10.3389/fimmu.2022.982264

**Published:** 2022-09-13

**Authors:** Yu-Jyun Li, Chi-Yuan Chen, Jeng-How Yang, Ya-Fang Chiu

**Affiliations:** ^1^Department of Microbiology and Immunology, Chang Gung University, Taoyuan, Taiwan; ^2^Graduate Institute of Biomedical Sciences, Chang Gung University, Taoyuan, Taiwan; ^3^Division of Infectious Diseases, Department of Medicine, Chang Gung Memorial Hospital, New Taipei, Taiwan; ^4^Research Center for Emerging Viral Infections, Chang Gung University, Taoyuan, Taiwan; ^5^Department of Laboratory Medicine, Linkou Chang Gung Memorial Hospital, Taoyuan, Taiwan

**Keywords:** influenza A virus, statins, inflammatory cytokines, lipid rafts, cholesterol

## Abstract

Influenza A virus (IAV) is widely disseminated across different species and can cause recurrent epidemics and severe pandemics in humans. During infection, IAV attaches to receptors that are predominantly located in cell membrane regions known as lipid rafts, which are highly enriched in cholesterol and sphingolipids. Following IAV entry into the host cell, uncoating, transcription, and replication of the viral genome occur, after which newly synthesized viral proteins and genomes are delivered to lipid rafts for assembly prior to viral budding from the cell. Moreover, during budding, IAV acquires an envelope with embedded cholesterol from the host cell membrane, and it is known that decreased cholesterol levels on IAV virions reduce infectivity. Statins are commonly used to inhibit cholesterol synthesis for preventing cardiovascular diseases, and several studies have investigated whether such inhibition can block IAV infection and propagation, as well as modulate the host immune response to IAV. Taken together, current research suggests that there may be a role for statins in countering IAV infections and modulating the host immune response to prevent or mitigate cytokine storms, and further investigation into this is warranted.

## Introduction

Influenza A virus (IAV) is a member of the *Orthomyxoviridae* family that causes seasonal outbreaks of respiratory infections in humans and animals. Influenza infections can induce life-threatening conditions such as pneumonia, particularly in the elderly (1, 2). Although influenza vaccines are available, even under the best conditions, when circulating viruses match the viral strains used to make vaccines, vaccination only reduces the risks of illness by 40% to 60% (3). This is because IAV mutates constantly, as the RNA-dependent RNA polymerase (RdRP) used in IAV viral RNA (vRNA) replication lacks proofreading activity (4, 5). Furthermore, genome reassortment across different species also generates IAV mutants that may be able to evade immune recognition and cause severe disease in hosts (6, 7). This constant changing of the virus poses a serious challenge to influenza vaccination prevention strategies (8). As for treatment after IAV infection, most mild to moderate infections are treated with a combination of over-the-counter drugs that can include antipyretics, analgesics, decongestants, antihistamines, and antitussives, which provide relief from symptoms but do not attack IAV or interfere with its propagation in any way (9). However, for treatment of severe cases, or for treatment or prophylaxis following confirmed exposure in groups at high risk of postinfection complications, antiviral medications that target the IAV life cycle can be used (10). These antivirals are summarized in **Table 1** and can be divided into five main classes: M2 protein inhibitors (amantadine and rimantadine), which disrupt the acidification of endosomes by blocking the M2 ion channel, thereby preventing the release of viral genomes to the cytoplasm (16); neuraminidase (NA) inhibitors (oseltamivir, zanamivir, laninamivir, and peramivir), which block the release of viral progeny by inhibiting NA activity (17); acidic endonuclease inhibitors (baloxavir marboxil), which inhibit the endonuclease activity required for viral gene transcription in the IAV RNA-dependent RNA polymerase (RdRP) complex (18); RNA polymerase inhibitors (favipiravir), which inhibit RdRP activity (19); and membrane fusion inhibitors (umifenovir), which block IAV from binding to and fusing with host cell membranes (15). However, new therapeutic approaches are constantly needed, as the high frequency of IAV mutation and reassortment drives resistance to treatment over time (20). One potential target is cholesterol (21, 22), which is abundantly present in areas known as lipid rafts on host cell membranes. Lipid rafts serve as hubs for cross talk and coordination of many regulatory and signaling events (23), and studies have shown that lipid rafts play important roles in the IAV life cycle; moreover, lipid rafts also serve as platforms for host immune responses. Interestingly, recent studies have shown that statins, a class of cholesterol-synthesis inhibitors, can act through cholesterol-dependent or -independent mechanisms to disrupt several stages of the IAV life cycle, as well as mediate host immune responses against IAV, and this may serve as a novel therapeutic approach to influenza treatment (21, 24, 25). In this review, the potential opportunities for statins at different stages of the IAV life cycle and host immune response are discussed, and current research is summarized to provide a comprehensive overview of the evidence surrounding the use of statins against IAV. Further research and examination of this topic is warranted, as statins do not target specific components of IAV, and mutational changes are less likely to confer resistance to treatment. This may mark an important paradigm shift in the decades-long arms race between humans and IAV.

**Table 1:** Antiviral drugs approved for use against influenza A viruses.

Class/drug	Side effects	Toxicity	Approval	Reference
**M2 protein inhibitors: Block the M2 ion-channel to prevent the release of viral genomes to the cytoplasm**				
Amantadine	Nausea, dizziness, insomnia	Central nervous system, sleep, gastrointestinal symptoms	US and multiple countries (now little used due to resistance)	11
Rimantadine	Insomnia, nausea, vomiting	Central nervous system, sleep, gastrointestinal symptoms	US and multiple countries (now little used due to resistance)	11
**Neuraminidase inhibitors: Inhibit neuraminidase activity to block the release of viral progeny**				
Oseltamivir	Nausea, vomiting, diarrhea	May correlate with sudden-onset type neuropsychiatric reactions	US and multiple countries	12
Zanamivir	Headaches, diarrhea, nausea	Central nervous system, psychiatric symptoms, gastrointestinal symptoms	US and multiple countries	13
Laninamivir	Abnormal behavior, diarrhea, nausea, dizziness	Central nervous system, psychiatric symptoms, gastrointestinal symptoms	Japan	10
Peramivir	Diarrhea, blurred vision, low neutrophil count	Central nervous system, gastrointestinal symptoms	US and multiple countries	10
**Acidic endonuclease inhibitors: Inhibit the endonuclease activity required for viral gene transcription in the viral RdRP complex**				
Baloxavir marboxil	Diarrhea, bronchitis, nasopharyngitis	Central nervous system, gastrointestinal symptoms	US and multiple countries	10
**RNA polymerase inhibitors: Inhibit RdRP activity**				
Favipiravir	Liver dysfunction, diarrhea, nausea	Embryotoxicity in animal studies	Japan	14
**Membrane fusion inhibitors: Block IAV from binding to and fusing with host cell membranes**				
Umifenovir	Drug sensitization and allergies	N/A	Russia, China	15

IAV, influenza A virus; N/A, not available; RdRP, RNA-dependent RNA polymerase; US, United States.

## Statins: Mechanism of action and antiviral properties

Statins, including atorvastatin, fluvastatin, lovastatin, pitavastatin, pravastatin, rosuvastatin, and simvastatin (26–31), are a class of drugs that block cholesterol synthesis through the inhibition of hydroxyl methylglutaryl-coenzyme A (HMG-CoA) reductase (26–31). Statins are now widely used in the primary and secondary prevention of cardiovascular disease (32). In addition, as the proliferation of many viruses requires cholesterol and cholesterol-rich lipid rafts on host cell membranes, the role of statins in countering viral infections has been examined in several studies (26, 28, 30, 31, 33). For instance, lovastatin has been shown to inhibit human immunodeficiency virus (HIV) entry to host cells by blocking the interaction between the cellular lymphocyte function-associated antigen-1 (LFA-1) receptor and intercellular adhesion molecule-1 (ICAM-1), which is present on the envelope of HIV viral particles. The inhibition by lovastatin decreases the propagation of HIV by 50% (28). Statins have also been reported to reduce the risk of severe COVID-19 by 70% (30). These findings suggest that statins may be useful in countering viral infections.

## Opportunities for disruption: The structure and life cycle of IAV

IAV is an enveloped virus that contains a genome consisting of eight single-stranded, negative-sense RNA segments (34). These segments encode at least 10 viral proteins, including hemagglutinin (HA); NA (35); the RdRP subunits PB1, PB2, and PA; nucleocapsid protein (NP); matrix proteins (M1 and M2); and non-structural proteins (NS1 and NS2) (1, 2, 36). The vRNA segments wrap around NPs and are bound by RdRP to form ribonucleoproteins (vRNPs), which are packaged within the virion. During infection, IAV is internalized by endocytosis through interactions between HA on the viral envelope with sialylated host receptors on the plasma membrane (37, 38). Following internalization of the virus, the low pH of the endosome environment activates M2 pH-gated proton channels on the viral envelope to acidify the viral interior, which in turn causes dissociation of the M1 matrix protein from the viral nucleoproteins (37, 38). The structure of HA is also altered inside the acidified endosomes, which leads to the fusion of the viral envelope with the endosomal membrane, followed by the release of vRNPs to the cytoplasm (37, 38).

**Figure 1:**
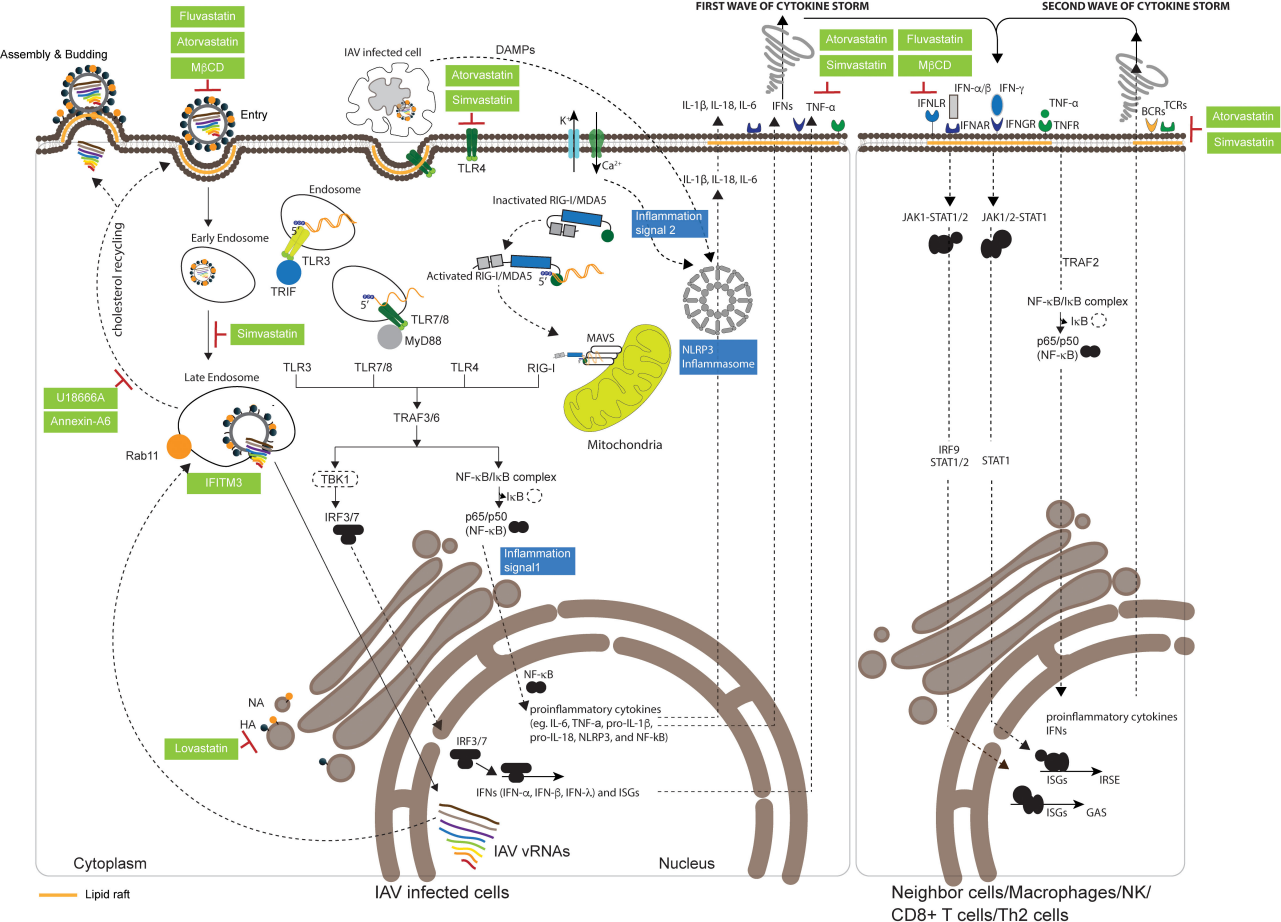
Effect of statins on the IAV life cycle and the host immune response. Influenza A virus (IAV) binds to sialylated host receptors at lipid rafts on the plasma membrane to initiate endocytosis for cellular entry. After internalization, acidification within the endosome causes the structural alteration of hemagglutinin (HA), which leads to the fusion of the viral envelope with the endosomal membrane and the release of viral genomes to the cytosol. Viral genomes are then transported to the nucleus for transcription and replication of viral RNA (vRNA). Several cholesterol biosynthesis regulators or inhibitors are known to be involved in the infection process: Methyl-β-cyclodextrin (MβCD) prevents IAV attachment; simvastatin reduces the efficiency of viral genome transport to the nucleus; fluvastatin and atorvastatin reduce IAV infectivity; and interferon-induced transmembrane protein 3 (IFITM3) inhibits the release of viral genomes from the endosome to the cytosol. Incoming single-stranded vRNA, double-stranded RNA intermediates that are formed during vRNA transcription and replication, and newly synthesized vRNA are recognized by immunosensors that can subsequently activate innate immunity, including toll-like receptor 3 (TLR3) and TLR7/TLR8 within the endosome, and retinoic acid-inducible gene I (RIG-I)/melanoma differentiation-associated gene 5 (MDA5), which interact with MyD88, TRIF, and mitochondrial antiviral signaling protein (MAVS) in the mitochondria. All TLRs and intracellular RIG-I initiating signals activate TANK-binding kinase 1 (TBK1) and the nuclear factor (NF)-kB/IkB complex, leading to the translocation of interferon regulatory factor-3 (IRF-3)/IRF-7 and NF-kB from the cytosol to the nucleus to induce the expression of interferons (IFNs), interferon-stimulated genes (ISGs), and proinflammatory cytokines. Following the maturation of proinflammatory cytokines induced by the nucleotide-binding oligomerization domain (NOD)-like receptor family pyrin domain (PYD)-containing 3 (NLRP3) inflammasome, inflammatory cytokines, tumor necrosis factors (TNFs), and IFNs are secreted extracellularly as the first wave of a cytokine storm. These secreted defense molecules are recognized by interferon-α receptors (IFNARs), interferon-γ receptors (IFNGRs), and TNF receptors (TNFRs) on neighboring cells, macrophages, natural killer (NK) cells, CD8^+^ T cells, or Th2 cells, which act to amplify innate immune signaling against IAV infection. Damage-associated molecular patterns (DAMPs) released from damaged or dying cells, including macrophages that take up the aggregated viral PB1-F2 protein, are recognized by TLR4. In addition, IAV M2 embedded in the *trans*-Golgi network (TGN) changes proton flux, which then activates NLRP3 complexes to induce the second wave of a cytokine storm. Atorvastatin, simvastatin, fluvastatin, and MβCD are capable of depleting or preventing the expression of IFNAR, IFNGR, and TNFR at lipid rafts, resulting in the stimulation of innate responses. T-cell receptors (TCRs) or B-cell receptors (BCRs), respectively presented at lipid rafts on T cells or B cells, are depleted by atorvastatin and simvastatin. IAV exploits the cholesterol recycling process to deliver newly synthesized vRNAs to lipid rafts on the plasma membrane for viral assembly through PB2–Rab11 interactions. U18666A and Annexin-A6, which retain and accumulate cholesterol in late endosomes to reduce the amount of cholesterol at lipid rafts, can decrease IAV production. Lovastatin, which depletes the cholesterol component on plasma membranes, prevents the trafficking of HA to the plasma membrane and alters the composition of cholesterol incorporated into viral particles.

After their release from virions, vRNPs are translocated to the nucleus, where their attached RdRP facilitate vRNA transcription and replication (36). IAV mRNA is then exported to the cytoplasm and translated into viral proteins (39). The vRNA is also used as a template for the synthesis of positive-stranded complementary RNAs (cRNAs), which are then used by RdRP as templates for vRNA replication (2, 40). The newly replicated vRNA is then packaged with NP and RdRP to form vRNPs, which are then exported to the cytoplasm through M1 and Rab-dependent recycling endosomes (37, 41, 42). HA and NA are also transported to lipid rafts, microdomains enriched with cholesterol, sphingomyelin, glycolipids, glycoproteins, and receptors on the host cell membrane, where they accumulate and facilitate viral particle assembly (**Figure 1**) (43–47). In the final stage, progeny viruses are assembled and released outside host cells by budding, and NA subsequently cleaves off sialic acid from the cellular receptor to prevent viral aggregation at the cell surface (37, 48, 49). Lipid rafts play an important role in the budding process, and the released virions are also coated in an envelope containing embedded cholesterol from the host cell membrane (43, 44). Given the prominent role of cholesterol and lipid rafts in the IAV life cycle, previous research has sought to identify and exploit opportunities where statins could make an impact on IAV infection and propagation.

## IAV internalization relies on lipid rafts

Many viruses are known to enter host cells through endocytosis and hijack endosomes for viral trafficking (50). It is known that IAV infection is mainly mediated by the binding of HA on the virion to clusters of sialyated glycoproteins or glycolipids on the cell surface (51–53). Although several glycoproteins, including epidermal growth factor receptor (EGFR), liver/lymph node-specific intracellular adhesion molecule-2 grabbing non-integrin (L-SIGN), and dendritic cell-specific intercellular adhesion molecule-3-grabbing non-integrin (DC-SIGN), have been shown to facilitate IAV attachment on cell surface for entry, whether these molecules are specific receptors for IAV uptake remain to be elucidated (54, 55). For example, a recent study has shown that IAV does not directly bind to EGFR, but the binding of IAV with multivalent sialic acid clusters can trigger EGFR activation (56). However, the glycoprotein carcinoembryonic antigen-related cell adhesion molecule 6 (CD66c or CEACAM6) has recently been identified as a receptor for IAV infection (57), and further research will undoubtedly identify more such interacting receptors in the future. After binding to cellular receptors, IAV is taken up by cells *via* either clathrin- or caveolin-dependent endocytosis, which usually occurs at lipid rafts (**Figure 1**) (50, 58). Lipid rafts serve as a platform for the cross talk and coordination of many regulatory proteins and signaling molecules, and cholesterol plays a key role in lipid raft structure and function. Several studies have shown that depletion of cholesterol from lipid rafts or viral envelopes with methyl-β-cyclodextrin (MβCD) prevents IAV attachment and reduces IAV infectivity; however, IAV infectivity is restored after exogenous cholesterol supplementation (**Figure 1**, **Table 2**) (55, 59–61). Moreover, sphingomyelin is known to coexist and interact with cholesterol on the plasma membrane (73), and disruption of sphingomyelin on lipid rafts with sphingomyelinases (SMase) also prevents IAV attachment and viral internalization (74, 75).

**Table 2:** Effects of molecules that deplete cholesterol, prevent cholesterol biosynthesis, or inhibit cholesterol trafficking, and ISGs on IAV infection.

Molecule/ISG	Functions	Effects on IAV	References
**Depletion of cholesterol**			
MβCD	Depletion of cholesterol from plasma membranes and viral envelopes	Prevention of IAV attachment for cellular entry Reduction of IAV infectivity	55, 59–61
**Prevention of cholesterol biosynthesis**			
Simvastatin	Prevention of RhoA prenylation	Inefficient transport of viral genomes into the nucleus for replication	62
Lovastatin	Reduction of cholesterol biosynthesis	Blocks HA trafficking to the plasma membrane Alteration of cholesterol composition on IAV	21, 25, 47
Fluvastatin/ atorvastatin	Reduction of cholesterol biosynthesis	Reduction of vRNA replication and viral protein synthesis	63, 64
**Inhibition of cholesterol transport**			
U18666A	Prevention of cholesterol shuttling from late endosomes to the plasma membrane	Reduction of IAV production	65–67
Annexin-A6	Prevention of cholesterol shuttling from late endosomes to the plasma membrane	Reduction of IAV production	65–67
Rab11	Complex formation with cholesterol Interaction with PB2 of vRNPs to deliver vRNPs to lipid rafts	Enhancement of cholesterol recycling for IAV packaging Facilitation of vRNP delivery to lipid rafts	68, 69
**ISGs regulating shuttle of cholesterol between organelles**			
IFITM3	Prevention of cholesterol trafficking from the ER to late endosomes	Inhibition of fusion pore formation to release IAV genomes to the cytosol	65, 70–72

ER, endoplasmic reticulum; HA, hemagglutinin; IAV, influenza A virus; ISG, interferon-stimulated gene; vRNA, viral RNA; vRNPs, viral ribonucleoproteins.

In addition, cholesterol is known to modulate IAV trafficking, and depletion of cholesterol disrupts IAV transport after cellular entry (60, 62, 64, 76). Mehrbod et al. (62) showed that simvastatin prevents RhoA prenylation, which is key to arrangement of the actin cytoskeleton for endosome trafficking, and results in inefficient transport of viral genomes into the nucleus for replication (**Figure 1**). Fluvastatin and atorvastatin treatment also reduces vRNA replication and viral protein synthesis (**Figure 1**) (63, 64). These findings show that cholesterol is critical to IAV binding and entry to host cells, and preliminary studies show that statins may be able to disrupt this process and prevent IAV proliferation (63, 64).

## IAV assembly and budding require lipid rafts

Following their glycosylation in the *trans*-Golgi network (TGN), HA and NA are transported to and accumulate at lipid rafts (**Figure 1**) (77, 78). An electron microscopy study showed that the cytoplasmic tails of HA and NA interact with the glycosphingolipid GM1 at lipid rafts, forming a structure called the budozone, where IAV budding occurs (44) (**Figure 1**). Substituting the amino acids in HA that are required for interaction with GM1 not only changes the amounts of HA on the viral particles but also reduces the number of IAV particles budding through lipid rafts (43).

Cholesterol is an essential component of lipid rafts (60), and the availability of cholesterol in lipid rafts has been shown to be critical to IAV replication. It has been shown that IAV production is significantly reduced after treatment with a cholesterol transport inhibitor, U18666A (**Figure 1**), and treatment of IAV-infected cells with Annexin A6, which causes retention and accumulation of cholesterol in the late endosomes to reduce cholesterol levels at lipid rafts, similarly reduces IAV production (**Figure 1**; **Table 2**) (65–67). During budding, cholesterol is also incorporated into the IAV envelope, constituting up to 44% of lipids, or 12% of the total mass of an IAV virion (60). Previous studies have shown that using lovastatin to reduce intracellular cholesterol and deplete lipid rafts not only influences the trafficking of HA to the plasma membrane but also alters the composition of cholesterol incorporated into the viral particles (**Figure 1**) (21, 25, 47).

Cholesterol in the endosomal membrane is acquired from the plasma membrane during endocytosis (79), and this endosomal cholesterol is recycled back to the plasma membrane by Rab11 (**Figure 1**) (80). IAV appears to exploit this cholesterol-recycling process to deliver vRNPs to lipid rafts on the plasma membrane for viral assembly, as PB2, a component of vRNP, has been shown to interact with the Rab11-cholesterol complex (**Figure 1**; **Table 2**) (68, 69). Furthermore, knockdown in the expression of Rab11 by shRNA, or expression of a mutant Rab11 that decreases the efficiency of cholesterol recycling back to the plasma membrane, was shown to reduce the amount of vRNPs conveyed to lipid rafts (**Figure 1**; **Table 2**) (68, 69). This indicates the importance of the cholesterol-recycling process to IAV maturation.

## Lipid rafts are important for IAV recognition by immunosensors

Many immunosensors, including toll-like receptors (TLRs) and c-type lectin receptors (CLRs), which are known as pattern recognition receptors (PRRs; 81), are localized at lipid rafts, where they detect viral infection and trigger defense machinery for viral clearance (**Figure 1**) (82, 83). In addition to PRRs on the plasma membrane, there are intracellular PRRs, such as RIG-I-like receptors (RLRs), which detect intracellular pathogens (84). After IAV infection, host cells utilize both types of PRRs to recognize either viral proteins or genomes and activate the immune response accordingly to defend against invasion (85, 86). Considering that the depletion of lipid rafts will not only decrease IAV production but also may affect immune responses against IAV infection (64, 87, 88), it is important to consider the role of statins in this context as well. Therefore, in this section, the main types of PRRs involved in IAV infection will be presented, and the impact of statins on PRRs and PRR-mediated immune responses will be discussed.

## Toll-like receptors

At least 10 different types of TLRs have been identified to date, all of which are capable of recognizing either extracellular or intracellular pathogen‐associated molecular patterns to trigger the secretion of proinflammatory cytokines and interferons (89–92). TLR1, TLR2, TLR4, TLR5, and TLR6 are present in lipid rafts on the cell surface, and their signaling is triggered by extracellular microorganisms and ligands (93–95). TLR3, TLR7, TLR8, and TLR9 are present on the cytosolic organelles and engage with either foreign ligands that are imported across plasma membrane barriers, or newly synthesized pathogenic components (85, 96, 97). However, some TLRs are found to shuttle between the plasma membrane and the cytosolic organelles to recognize incoming pathogens (95). For instance, TLR3 and TLR9 are transported to the cell surface to recognize extracellular pathogens, while cytosolic forms of TLR2 and TLR4 are found in the cytoplasm, TGN, endoplasmic reticulum (ER), or nucleolus, where they are known to engage the HA of measles virus, the glycoprotein B (gB) of herpes simplex virus (HSV), the F protein of respiratory syncytial virus (RSV), or the envelope protein of mouse mammary tumor virus (MMTV) (97–99).

After internalization of IAV through endocytosis, vRNPs are released to the cytosol following the acidification of endosomes and fusion of the viral envelope with endosomal membranes (100). The IAV genome is recognized by several TLRs, including TLR3, TLR7, and TLR8 (**Table 3**) (85, 96, 108), among which endosomal TLR7 and TLR8 serve as the first immunosensors for incoming vRNPs (96). TLR7 recruits MyD88 to induce proinflammatory cytokines and chemokines such as tumor necrosis factor-α (TNF-α), interleukin-6 (IL-6), and IL-1β and can further promote type I interferon release, dendritic cell (DC) maturation, and antiviral immunity (**Figure 1**) (109, 110). In addition, TLR3 senses IAV double-stranded RNA (dsRNA) intermediates formed during IAV transcription and vRNA replication within the endosome, and binding of the dsRNA to TLR3 triggers the expression of IFN-β and proinflammatory cytokines (**Figure 1**) (85). As TLR3-defective mice produce significantly few cytokines after IAV infection, TLR3 is believed to play a critical role in IAV clearance (111). In contrast to TLR7 and TLR3, TLR4 is present on the cell surface and detects IAV infection by recognizing S100A9, which is a damage-associated molecular pattern (DAMP) secreted by macrophages after IAV infection (**Figure 1**) (108). Preliminary evidence shows that depletion of TLR4 from lipid rafts by atorvastatin and simvastatin may reduce IAV clearance (**Figure 1**; **Table 3**) (101, 102), although further research is needed to better understand the extent of such effects.

**Table 3:** Immunosensors and receptors embedded in lipid rafts for IAV recognition.

Immunosensors	Recognition	Statins	Effects	References
**TLRs**				
TLR3	dsRNA (IAV)	N.D.	N.D.	85
TLR4	DAMPs (IAV-infected cells)	Atorvastatin, simvastatin	Depletion of TLR4 from lipid rafts by atorvastatin	101, 102
TLR7/8	ssRNA (IAV)	N.D.	N.D.	96
**CLRs**				
DC-SIGN/L-SIGN	IAV	N.D.	N.D.	54
Langerin	IAV	N.D.	N.D.	103
**TNFRs**				
TNFR1	TNF-α	N.D.	N.D.	104
**IFNARs**				
IFNAR1	Type I interferon	Simvastatin, atorvastatin	Prevention of IFNAR1 expression and endocytosis for initiating innate immune responses	105
IFNGR	Type II interferon	MβCD	Depletion of IFNGR in lipid rafts	106
IFNLR	Type III interferon	N.D.	N.D.	107

CLRs, C-type lectin receptors; DAMPs, damage-associated molecular patterns; DC-SIGN, dendritic cell-specific ICAM3-grabbing non-integrin; dsRNA, double-stranded RNA; IAV, influenza A virus; IFNAR, interferon-α receptor; IFNGR, interferon-γ receptor; IFNLR, interferon-λ receptor; L-SIGN, liver/lymph node-specific intercellular adhesion molecule-3-grabbing integrin (also known as DC-SIGN-R); MβCD, methyl β-cyclodextrin; N.D., not determined; ssRNA, single-stranded RNA; TLRs, toll-like receptors; TNF-α, tumor necrosis factor-α, TNFRs, tumor necrosis factor receptors.

## C-type lectin receptors

Another group of immunosensors in lipid rafts that are known to influence IAV infection are CLRs, which are transmembrane glycoproteins expressed by monocytes, macrophages, DCs, and Langerhans cells (LCs; 112–114). CLRs are recruited to lipid rafts and function as PRRs against the glycans of glycoproteins on pathogens (115). CLRs have been found to be important for IAV infection (54, 103), and several studies have shown that CLRs such as DC-SIGN, L-SIGN (also known as DC-SIGN-R), and langerin can facilitate IAV infection (**Table 3**) (54, 103). Londrigan et al. (54) showed that IAV is recognized and internalized through DC-SIGN and L-SIGN on Lec2 Chinese hamster ovary cells, which lack sialic acid-modified glycoproteins that are usually recognized by IAV and mediate infection. Moreover, Ng et al. (103) showed that the internalization of IAV is mediated through the binding of HA to langerin, suggesting that CLRs can function as receptors for IAV infection. The effect of statins on CLRs remains unclear, and further research is needed to ascertain if statins can interact with CLRs and disrupt their facilitating effect on IAV infection.

## Intracellular sensors

In addition to PRRs that are embedded on the cell surface, many intracellular soluble PRRs such as NOD-like receptors (NLRs) recognize IAV to trigger innate immunity (81, 116, 117). At the early stage of IAV infection, proteins encoded by interferon-stimulated genes (ISGs), such as serine/threonine kinase protein kinase R (PKR), trigger the formation of stress granules when newly synthesized uncapped vRNA and retinoic acid-inducible gene-I (RIG-I) are recruited (118). Following the recognition of uncapped vRNA, RIG-I is activated and subsequently induces the polymerization of mitochondrial antiviral signaling protein (MAVS) on the outer mitochondrial membrane (**Figure 1**) (119). As MAVS preferentially oligomerizes at the sites of mitochondria with high cholesterol content (120), this oligomerization may be modulated by cholesterol as well. RIG-I-MAVS signaling leads to either the activation of nuclear factor-κB (NF-κB) through TNF receptor-associated factor (TRAF)-3, TRAF-6, and receptor-interacting protein 1 (RIP-1) or the phosphorylation and activation of interferon regulator factor (IRF)-3 and IRF-7 *via* TRAF3 (121–124). Activated IRF-3/IRF-7 and NF-κB subsequently translocate to the nucleus to activate the expression of IFNs, ISGs, and proinflammatory genes, including those encoding IL-1β and IL-18 (**Figure 1**) (117, 123, 125, 126). Following secretion, IFNs bind to the receptors on the cell surface of IAV-infected cells or their neighbor cells to activate the JAK-STAT pathway, which induces the expression of ISGs to act against IAV infection (**Figure 1**) (127, 128). Similar to RIG-I, melanoma differentiation-associated gene 5 (MDA5) is also activated by IAV dsRNA intermediates and then recruited to the outer mitochondrial membrane to trigger the IRF-3/IRF-7 and NF-κB signaling pathway, which in turn promotes the expression of IFN, ISGs, and proinflammatory genes (**Figure 1**) (129–133).

Similarly, two major NOD-like receptor (NLR) molecules, nucleotide-binding oligomerization domain 2 (NOD2) and NOD-, leucine-rich repeat (LRR)-, and pyrin domain-containing protein 3 (NLRP3), serve as PRRs upon IAV infection (134, 135). NOD2 recognizes IAV single-stranded RNA (ssRNA) and triggers the activation and translocation of IRF-3/IRF-7 and MAPK, by respectively recruiting the adaptor proteins, MAVS and RIPK2, to induce IFN-α/β and proinflammatory cytokine production (135); NLRP3 is also a critical component of the inflammasome, which induces the secretion of IL-1β and IL-18 and triggers a cytokine storm upon IAV infection (**Figure 1**) (134, 136).

Recognition of incoming or newly synthesized IAV genomes by either transmembrane or intracellular PRRs activates innate immunity and initiates the expression of first-wave IFNs, ISGs, chemokines, and proinflammatory cytokines, which stimulate immune cell infiltration to activate the expression of second-wave cytokines for IAV clearance. However, innate immunity may cause uncontrolled and excessive release of inflammatory cytokines to result in acute respiratory distress syndrome (ARDS), also known as a cytokine storm (**Figure 1**) (137) (Please also see the section, “IAV-induced inflammation and cytokine storms”). Lipophilic statins have been reported to exert a number of pleiotropic effects on the NLRP3 complex, acting to reduce inflammatory activity (138). This may have an effect in preventing or mitigating cytokine storms, which are a major cause of morbidity and mortality in severe influenza infections (139).

## Immunosensor activation of the immune response against IAV

Immunosensors stimulated by infection trigger signaling to activate the expression of IFNs, TNFs, cytokines, and ISGs (140–143). These defense molecules then interact with immunoreceptors, including TNF-α receptors (TNFRs) and interferon receptors (IFNRs), to induce immunosignaling cascades against pathogens (127, 144).

## Tumor necrosis factor receptors

TNF-α is a proinflammatory cytokine that is upregulated after IAV infection (145) and is secreted from infected cells to trigger warning signals in neighboring cells *via* binding to TNFR1 or TNFR2 on cell surfaces (**Figure 1**; **Table 3**) (146). The secreted TNF-α also attracts immune cells and stimulates infiltration (147). In addition, release of TNF-α from vesicles through fusion with the plasma membrane is mediated by the N-ethylmaleimide-sensitive factor attachment protein receptor (SNARE) complex, which is enriched at lipid rafts (148). MβCD treatment to deplete cholesterol at lipid rafts reduced the secretion of TNF-α, and Legler et al. (104) further showed that TNFR1 translocates to lipid rafts, where it associates with the serine/threonine kinase RIP, TRADD, and TRAF2 as a signaling complex. Depletion of lipid rafts by MβCD abrogates TNF-α-mediated NF-κB activation, suggesting that TNFR1 assembly at lipid rafts is essential for NF-κB activation during IAV infection. Embedded TNFR2 in the lipid rafts of CD8^+^ T cells is essential for interaction with TNF-α and induction of the immune response against IAV infection; however, excessive TNFR2 expression can lead to cytokine storms that may cause severe and lethal lung injury (149).

## Interferon receptors

Three types of interferon receptors, IFN-α receptors (IFNARs), IFN-γ receptors (IFNGRs), and INF-λ receptors (INFLRs), can recognize their respective IFNs when these are released from immune cells or pathogen-infected cells (**Figure 1**; **Table 3**) (150–152). IFN-α/β is expressed in immune cells, including macrophages, alveolar cells, DCs, and inflammatory monocytes, while IFN-γ is expressed by NK cells and cytotoxic T cells (CTLs) (153–155). This recognition process triggers immunopathology during a cytokine storm. IFNLRs are present on mucosal epithelial cells and recognize IFN-λ, which is typically released from myeloid cells, epithelial cells, and DCs (**Figure 1**) (107). Reduction of cholesterol with MβCD is known to disrupt localization and assembly of IFNGR at lipid rafts (106), while depletion of cholesterol by simvastatin and atorvastatin prevents IFNAR1 expression and endocytosis (**Figure 1**; **Table 3**) (105).

During IAV infection, IFNs are produced and released from IAV-infected cells after recognizing IAV ssRNA or dsRNA (85, 109), and when these IFNs bind with their respective receptors, both IFNARs and IFNGRs are recruited to lipid rafts and internalized through endocytosis (**Figure 1**) (156). This interaction induces the recruitment and phosphorylation of the JAK-STAT and tyrosine kinase 2 (TYK2) pathways (150, 157). Following the recruitment and autophosphorylation of STAT1/2 (158), phosphorylated STAT1/2 forms a transcription factor complex with IRF-9, termed IFN-stimulated gene factor 3 (ISGF3; 159), which translocates from the cytosol to the nucleus and binds to IFN-stimulated response elements (ISREs) in the ISG promoters to initiate the transcription of genes against viral infection (160, 161). Among ISGs activated by viruses, IFN-induced transmembrane protein 3 (IFITM3) has been reported to restrict the replication of dengue virus, West Nile virus, coronavirus, and IAV (72, 162). In the early stages of IAV infection, IFITM3 is upregulated after activation by IFN signaling (**Figure 1**) (**Table 2**). IFITM3 prevents the transport of cholesterol from ER to late endosomes, thus affecting fusion with the IAV envelope, and also blocks the formation of fusion pores to disrupt the release of vRNPs (**Figure 1**; **Table 2**) (65, 70–72). Several recent studies have shown that statins can inhibit IFN signaling and activity (87, 105), likely through the inhibition of IRF-3 and JAK/STAT signaling in macrophages (105), and the impact of this on the host antiviral response is worthy of further investigation. Interestingly, a study of gammaherpesvirus infection showed that type I interferon counters the antiviral effects of statins derived through the reduction of cholesterol, and therefore the reported inhibition of IFN activity by statins may be expected to enhance their cholesterol-dependent antiviral activity (163).

## Lipid rafts serve as a platform for the host adaptive immune response against IAV

Although innate immune responses are known to limit IAV replication and transmission (86, 109, 145, 164), IAV clearance requires substantial activation, clonal expansion, recruitment, and acquisition of effector immune cells at the respiratory tract, as part of the adaptive immune response (165–167). However, activated adaptive immunity can lead to excessive inflammatory responses that are prone to induce cytokine storms and cause severe or fatal lung injury (168, 169). Therefore, a well-controlled adaptive immune response is essential to avoid triggering cytokine storms (170, 171).

After IAV infection, B-cell receptors (BCRs) on mature B cells interact with antigens presented on antigen-presenting cells (APCs) and then translocate to lipid rafts, where they recruit co-stimulatory factors to trigger downstream activating signals (**Figure 1**) (172–174). Antigens recognized by BCR are then internalized through BCR-mediated endocytosis and processed within major histocompatibility complex class II (MHCII)-containing lysosomes; ultimately, the processed antigens are presented on the cell surface (172–174). The presented antigens on the MHCII of B cells are recognized by T-cell receptors (TCRs) of CD4^+^ T cells, leading to expression of the surface protein CD40L, as well as the cytokines IL-4 and IL-21, to enable activation of B cells *via* the interaction with CD40 and cytokine receptors on B cells (175, 176). In this way, B cells present antigens to stimulate CD4^+^ T cells, and this in turn enables the activation of B cells and the synthesis of antigen-specific antibodies (177, 178). In the case of IAV infection, the assistance from CD4^+^ T cells enables B cells to mature as plasmablasts (PBs) at germinal centers (GCs), and the matured B cells then produce antibodies targeting the surface HA or NA on IAV virions, thereby preventing IAV infection or egress (179–181). Mature B cells also produce anti-M2 antibodies to prevent IAV production (182). Moreover, secreted antibodies against IAV can also serve to mediate antibody-dependent cell-mediated cytotoxicity (ADCC) through NK cells, macrophages, γδ T cells, and leukocytes (183, 184).

In addition to antibodies generated by B cells, T cells can also play a critical role in preventing IAV infection (165). T cells differentiate in the thymus into CD4^+^ T and CD8^+^ cells (185), which subsequently differentiate further into cytotoxic T cells (CTLs) after recognizing IAV-associated antigens presented on major histocompatibility complex class I (MHCI) molecules on DCs (185). Activated CTLs recognize IAV-infected cells, and in response, they produce cytokines (TNF-α/β, IFN-γ, and IL-2) and cytotoxic granules containing granzymes and perforin, which induce the formation of pores on CTL-targeted cells and restrict IAV replication (186). CTLs also induce apoptosis of IAV-infected cells by delivering granzymes through perforin-mediated pores and secreting cytokines such as TNF, Fas ligand (FasL), and TNF-related apoptosis-inducing ligand (TRAIL) to recruit death receptors (187, 188); however, CTL infiltration in respiratory tracts often causes excessive production of proinflammatory cytokines by respiratory cells or immune cells recruited to the airways, and severe injury of lung tissues may follow as a result (168, 189). IFN-γ and TNF-α secreted by CD8^+^ T cells are known to enhance the release of lung epithelial chemokines, which promote inflammatory cell infiltration, lung pathogenic injury, and apoptosis of IAV-infected or non-infected lung epithelial cells, thereby raising the risk of severe cytokine storms (168, 189, 190). It has been shown that treatment with anti-IFN-γ significantly reduced lung pathology, inflammatory cell infiltration, and mortality of mouse models infected by IAV, indicating that IFN-γ is a key molecule involved in the development of a cytokine storm. Statins are known to inhibit the production of several inflammatory cytokines (87, 102, 105) and may serve to modulate the cytotoxic activity of CD8^+^ T cells during IAV clearance. Unlike CD8^+^ T cells, CD4^+^ T cells are activated by antigens presented on MHCII molecules, followed by the binding of CD40L to CD40 on APCs such as DCs (191). Activated CD4^+^ T cells facilitate B-cell activation and antibody production (192) and can also differentiate into various subtypes, depending on the co-stimulatory cytokines received from the microenvironment (193–196).

Similar to BCRs, TCRs are located at non-raft regions during the resting state but are translocated to lipid rafts following T-cell activation (**Figure 1**) (197, 198). After TCRs recognize antigens presented on MHCs present on APCs, TCRs interact with the CD4- or CD8-lymphocyte-specific protein tyrosine kinase complex (199) to initiate the T-cell activation signaling cascade (200, 201). Failure of the Src-family kinase Lck to localize to lipid rafts (202), depletion of lipid rafts by atorvastatin (203), or reduction of intracellular cholesterol by simvastatin and atorvastatin (204) can abort the activation of adaptive immune responses. During the activation of T and B cells, several co-stimulatory factors, including CD40, CD83, and CD86, also localize to lipid rafts and are required for T-cell and B-cell activation (205, 206). Shimabukuro-Vornhagen et al. (204) showed that simvastatin and atorvastatin inhibit B-cell activation and proliferation by downregulating the expression of CD40 and other co-stimulatory factors such as CD80 and CD86, and MHCII, in a dose-dependent manner. Additionally, statins can reduce the expression of CD40, CD83, and CD86, as well as the secretion of IL-6, IL-8, IL-12, and TNF-α by DC, resulting in the inhibition of DC-induced T-cell proliferation and activation (207). These studies show that lipid rafts serve as a platform to regulate adaptive immune responses (173, 197, 206, 208), and the modulation of lipid rafts with statins may represent a promising approach to manage the adaptive immune response to IAV infection.

## IAV-induced inflammation and cytokine storms

Inflammation is an innate immune response that protects cells from IAV infection (134, 209). Inflammasome formation is tightly regulated by two sequential signals, a priming signal and an activating signal (210–214). Inflammasome components (e.g., NLRP3) and proinflammatory cytokines are upregulated by NF-κB signaling *via* TLRs, RLRs, TNFR1, and IL-1 receptors, which serve as a priming signal (**Figure 1**) (210, 215, 216). DAMPs released from damaged or dying cells infected by IAV are then sensed by NLRP3, and this triggers the activating signal (**Figure 1**) (108, 134). A variety of molecules can serve as DAMPs, including IAV ssRNAs that stimulate the release of IL-1β, IAV M2 embedded in the TGN that can alter proton flux, and aggregated PB1-F2 derived from dying infected cells that are taken up by macrophages and transported to lysosomes (136, 217). Activated NLRP3 recruits an adaptor, ASC (apoptosis-associated speck-like protein containing a caspase recruitment domain (CARD), also known as PYCARD), along with caspase 1, to activate inflammatory caspases and promote the maturation of IL-1β and IL-18 (**Figure 1**); this process also stimulates pyroptosis, which is a rapid, inflammatory form of lytic programmed cell death induced after infections (217–221).

Following internalization of IAV by host cells, the virus is recognized by TLRs, CLRs, or RLRs, which initiate the innate immune response within infected cells to release cytokines (81). These cytokines are in turn recognized by NK cells, CD8^+^ T cells, Th2 cells, macrophages, and neutrophils, resulting in the stimulation and production of a second wave of cytokines, which ultimately enhance the release of lung epithelial chemokines (168, 222–225) (Please also see the section, “Lipid rafts serve as a platform for the host adaptive immune response against IAV”). The chemokines aggravate apoptosis of lung cells but can also increase inflammatory cell infiltration to promote IAV clearance (154, 226). However, when the production of proinflammatory cytokines, including TNF-α, IFN-α/β, IL-6, and IL-1β, becomes excessive and spirals out of control, or if anti-inflammatory factors fail to curb the growing inflammatory response, a cytokine storm develops (**Figure 1**). The condition often causes severe or fatal lung injury (168, 169, 227, 228). Therefore, cytokine dysregulation is regarded as a major pathophysiological mechanism in IAV infection, with potentially fatal consequences, as seen in the 1997 H5N1 Hong Kong avian influenza epidemic and the 1918 influenza pandemic (229, 230). Inhibition of the excessive inflammatory responses driven by a cytokine storm is considered to be an effective approach in preventing fatal IAV infections (231, 232). Several therapies, including the TNF-α inhibitor etanercept, the sphingosine analog AAL-R, and tyrosine kinase inhibitors such as ponatinib, have been assessed for efficacy in blocking IAV-induced cytokine storms (233–237). Statins have also been shown to inhibit the production of inflammatory cytokines, including TNF-α, IFN-γ, and IL-6 or IL-8 (62, 64), and these effects may have utility in preventing cytokine storms and fatal IAV infections (**Figure 1**) (234).

## The clinical potential of statins in IAV treatment

Statins have been shown to prevent IAV propagation and transmission in cell culture and animal studies (62, 63, 209), suggesting that there may be a role for statins in the treatment of IAV infection. Several studies show benefits of statin use during IAV infection, including a 40% reduction in the risks of pneumonia death caused by IAV infection (238), reduction of fatal IAV infection cases in hospitalized IAV-infected inpatients during the 2007–2008 epidemic season (239), and reduced risk of developing influenza-associated pneumonia in patients regularly taking statins to prevent cardiovascular disease (240). This was also noted in a study by Brassard et al. (241), who analyzed the records of approximately 10,000 patients in the UK Clinical Practice Research Datalink and found that regular use of statins significantly lowered hospitalization and mortality rates during IAV infection. Recently, a meta-analysis on statin efficacy in IAV infection also found that the use of statins significantly reduced influenza prevalence among both flu-vaccinated and unvaccinated subjects and was associated with significantly reduced mortality after IAV infection, including both 30- and 90-day mortality after diagnosis of infection (242). This suggests that statins can indeed have a positive clinical impact on both preventing IAV infection and mitigating the severity of disease after infection.

However, many other studies have failed to confirm that statins provide substantial protection from IAV infection. In a retrospective cohort study that examined patients from administrative healthcare databases in Ontario from a 10-year period (1996 to 2006), statins were found to provide slight protective effects against IAV-induced pneumonia hospitalization, 30-day pneumonia mortality, and all-cause mortality among approximately 2 million people aged 65 and older (243). A single-center retrospective study investigating statin uses and outcome in hospitalized patients during the 2009 influenza pandemic found that the use of statins lowered the number of cases with severe or lethal lung injury, but the benefits of statin treatment on the reduction of fatal infections was not statistically significant (244). Izurieta et al. (245) analyzed about 1,400,000 patients prescribed with statin treatment but did not find any benefits regarding IAV infection. Cutrell et al. (246) also did not find any positive correlation between statin usage and the reduction of acute illness caused by IAV infection. Similarly, Brett et al. (247) found that statins had no effect on the reduction of severe illness caused by IAV infection.

In light of the complex factors involved in these observational clinical studies, Izurieta et al. (245) studied whether statins could be used against IAV infection in mice under well-controlled etiology and pathology conditions. However, the study showed that statins provided only marginal inhibitory effects on protection from IAV infection. In a similar mouse model system, Belser et al. (248) showed that simvastatin reduced levels of IFN-γ, IL-10, and TNF-α, all cytokines known to be involved in lung infiltration, but the survival rate of the mice did not increase after infection. In a similar study, Radigan et al. (249) found that rosuvastatin did not increase the survival of infected experimental animals. The lack of increase in survival rates following treatment might be due to the use of high titers of IAV in these studies, which caused rapid death in the animal models studied and may not have allowed sufficient exploration of the benefits of statins against IAV infection.

## Future perspectives

The inconsistency of clinical benefits for statin use in the treatment of severe influenza infection may be multifactorial, being partly due to the variation in timing and duration of statin administration between participants, and partly due to the differences in pathogenic mechanism(s) of pneumonia induced by viral, bacterial, or other pathogens, course of acute respiratory infection, vaccination against IAV infection, and other risk factors, such as chronic or cardiovascular diseases (241). Moreover, statins may also affect innate and adaptive immunity (102, 203), and thus any antiviral benefit from statin treatment may be offset by these effects on the innate immune response. Therefore, comprehensive observational studies on individuals who do not regularly use cardioprotective statins or immunomodulatory agents should be conducted, to provide better information on the therapeutic potential of statins. Interestingly, Karlsson et al. (250) found that simvastatin reduced symptoms of IAV-induced pneumococcal pneumonia in obese mice, suggesting that lipid metabolic status may influence the protective capability of statins in IAV-associated respiratory diseases.

From another perspective, the triggering of cytokine storms can be influenced by age, gender, and pregnancy (251). The risk of developing secondary bacterial infections after IAV infection ranges from 2% to 65% (252) and is closely associated with obesity, as excess lipids in obese individuals can increase the number of leukocytes and monocytes in the blood, upregulate activating interactions between B cells and T cells, and raise the number of Th1 and Th17 cells (253). These changes often lead to chronic cell infiltration and inflammation, which can heighten the risk of cytokine storms during IAV infection (254). A phase 2 clinical trial (ClinicalTrials.gov Identifier: NCT02056340) conducted from October 2013 to June 2018 at Beth Israel Deaconess Medical Center showed that atorvastatin treatment of inpatients diagnosed with IAV infection but without statin pretreatment or liver- or cardiovascular-associated diseases significantly reduced levels of the inflammatory cytokine IL-6, which may help to prevent the occurrence of cytokine storms (30). These promising findings offer hope regarding the use of statins to prevent excess mortality in IAV pandemics.

## Concluding remarks

There is no solid clinical evidence to support the benefits of treating severe influenza illness with statins as yet, but data from some observational cohorts suggest that statin therapy is associated with a reduction in poor outcomes and mortality. The efficacy of statins in influenza management should be examined in larger double-blind, placebo-controlled, and randomized trials for hospitalized statin-naïve patients with IAV infection, and the metabolic status of patients should be taken into account as a key variable in future studies. Clinicians should also be mindful of the effects on immunity when weighing the benefits and risks of prescribing statins to patients.

## Data availability statement

The original contributions presented in the study are included in the article/supplementary materials. Further inquiries can be directed to the corresponding author/s.

## Author contributions

Conception and design: All authors. Literature analysis and interpretation: All authors. Writing the manuscript: All authors, and Y-JL and C-YC contributed equally to this work. Final approval: All authors. All authors contributed to the article and approved the submitted version.

## Funding

This work was financially supported by the Ministry of Science and Technology (MOST), Taiwan (MOST 109-2320-B-182-028-MY3); the Chang Gung Medical Research Program (CMRPD1K0321 and CMRPD1K0322); Chang Gung Memorial Hospital, Linkou (BMRPF14); and the Research Center for Emerging Viral Infections from The Featured Areas Research Center Program within the framework of the Higher Education Sprout Project by the Ministry of Education (MOE) and MOST in Taiwan (MOST 110-2634-F-182-001, MOST 109-2327-B-182-002).

## Acknowledgments

We thank Dr. Chih-Ho Lai for his insights and suggestions.

## Conflict of interest

The authors declare that the research was conducted in the absence of any commercial or financial relationships that could be construed as a potential conflict of interest.

## Publisher’s note

All claims expressed in this article are solely those of the authors and do not necessarily represent those of their affiliated organizations, or those of the publisher, the editors and the reviewers. Any product that may be evaluated in this article, or claim that may be made by its manufacturer, is not guaranteed or endorsed by the publisher.
